# Account of Diseases in an Eastern District of London

**Published:** 1802-11-01

**Authors:** 


### *Account of Diseases in an Eaftern Diftrict of London.*

From September 20 to October 20, 1802.

| | | | |
|---|---|---|---|
| ACCUTE DISEASES. | | Dyfenteria - - - - | 12 |
| Typhus - - - - - - - | 7 | Variola - - - - - - - | 2 |
| Cholera - - - - - - | 30 | Rheumatifmus Acutus - | 5 |

CHRONIC

| | | | |
|---|---|---|---|
| **Chronic Diseases.** | | Menorrahagia | 3 |
| Tuſſis | 7 | Fluor Albus | 9 |
| Dyſpnœa | 3 | Amenorrhœa | 5 |
| Tuſſis cum Dyſpnœa | 9 | Dyſuria | 3 |
| Hæmoptyſis | 4 | Calculus | 1 |
| Hydrothorax | 2 | Rheumatiſmus Chronicus | 10 |
| Anaſarca | 4 | **Puerperal Diseases.** | |
| Diarrhœa | 16 | Menorrhagia Lochialis | 4 |
| Enterodynia | 7 | Lochiorum Diminutio | 2 |
| Cephalalgia | 4 | Maſtodynia | 5 |
| Vertigo | 3 | Rhagas Papillæ | 2 |
| Paralyſis | 1 | **Infantile Diseases.** | |
| Syphilis | 3 | Ophthalmia | 4 |
| Hepatitis Chronica | 2 | Vermes | 3 |
| Herpes | 4 | Tinea Capitis | 2 |
| Hæmorrhois | 3 | Pertuſſis | 4 |

The diſeaſes which formed ſo large a proportion of the liſt publiſhed in the laſt month, ſtill continue to engage a conſiderable ſhare of medical attention. Diſorders of the ſtomach and bowels have been conſidered as the prevailing epidemic of the laſt two months. The number of theſe complaints is, however, diminiſhed; and the ſymptoms, for the moſt part, appear under a milder form. Moſt of the caſes of cholera, which have occurred ſince the laſt Report, have been attended with a mild train of ſymptoms.

The diluting and demulcent plan of treatment, with the occaſional uſe of opiates, has been employed with ſucceſs.

The dyſentery has, in ſome inſtances, been diſtinguiſhed by a very conſiderable diſcharge of blood; and in theſe caſes, it has proved contagious, as more than one in a family have been affected by it. In one inſtance the diſeaſe was attended with aphthous ulcerations of the mouth and throat. Suppreſſion of urine alſo, in an elderly man was an additional ſource of diſtreſs and pain. Severe gripings preceding the different diſcharges from the rectum, and a teneſmus immediately following them conſtitute, a conſiderable part of the patient's ſufferings. This diſeaſe not being diſtinguiſhed in the early ſtage of it from a diarrhœa, has too frequently led to a very injurious plan of treatment. Nothing is more common than to find that the patient has been very induſtrious in the uſe of aſtringents to check the diſcharge, and of cordials to ſupport his ſtrength under the weakneſs which he ſuppoſes it has produced. Inſtead of thus attempting to prevent the evacuation, which, though it is productive of great uneaſineſs, is very trifling in quantity, the more ſucceſsful mode of treatment is that of quieting the irritation of the ſtomach if it be neceſſary, removing ſtricture in the large inteſtines, and by ſome active cathartics promoting a thorough evacuation from the inteſtinal canal.

